# Remodeling of Purinergic Receptor-Mediated Ca^2+^ Signaling as a Consequence of EGF-Induced Epithelial-Mesenchymal Transition in Breast Cancer Cells

**DOI:** 10.1371/journal.pone.0023464

**Published:** 2011-08-05

**Authors:** Felicity M. Davis, Paraic A. Kenny, Eliza T-L. Soo, Bryce J. W. van Denderen, Erik W. Thompson, Peter J. Cabot, Marie-Odile Parat, Sarah J. Roberts-Thomson, Gregory R. Monteith

**Affiliations:** 1 School of Pharmacy, The University of Queensland, Brisbane, Queensland, Australia; 2 Department of Developmental and Molecular Biology, Albert Einstein College of Medicine, New York, New York, United States of America; 3 St. Vincent's Institute, Fitzroy, Victoria, Australia; 4 University of Melbourne Department Surgery, St. Vincent's Hospital, Fitzroy, Victoria, Australia; 5 University of Melbourne Department Medicine, St. Vincent's Hospital, Fitzroy, Victoria, Australia; Baylor College of Medicine, United States of America

## Abstract

**Background:**

The microenvironment plays a pivotal role in tumor cell proliferation, survival and migration. Invasive cancer cells face a new set of environmental challenges as they breach the basement membrane and colonize distant organs during the process of metastasis. Phenotypic switching, such as that which occurs during epithelial-mesenchymal transition (EMT), may be associated with a remodeling of cell surface receptors and thus altered responses to signals from the tumor microenvironment.

**Methodology/Principal Findings:**

We assessed changes in intracellular Ca^2+^ in cells loaded with Fluo-4 AM using a fluorometric imaging plate reader (FLIPR^TETRA^) and observed significant changes in the potency of ATP (EC_50_ 0.175 µM (−EGF) versus 1.731 µM (+EGF), *P*<0.05), and the nature of the ATP-induced Ca^2+^ transient, corresponding with a 10-fold increase in the mesenchymal marker vimentin (*P*<0.05). We observed no change in the sensitivity to PAR2-mediated Ca^2+^ signaling, indicating that these alterations are not simply a consequence of changes in global Ca^2+^ homeostasis. To determine whether changes in ATP-mediated Ca^2+^ signaling are preceded by alterations in the transcriptional profile of purinergic receptors, we analyzed the expression of a panel of P2X ionotropic and P2Y metabotropic purinergic receptors using real-time RT-PCR and found significant and specific alterations in the suite of ATP-activated purinergic receptors during EGF-induced EMT in breast cancer cells. Our studies are the first to show that P2X_5_ ionotropic receptors are enriched in the mesenchymal phenotype and that silencing of P2X_5_ leads to a significant reduction (25%, *P*<0.05) in EGF-induced vimentin protein expression.

**Conclusions:**

The acquisition of a new suite of cell surface purinergic receptors is a feature of EGF-mediated EMT in MDA-MB-468 breast cancer cells. Such changes may impart advantageous phenotypic traits and represent a novel mechanism for the targeting of cancer metastasis.

## Introduction

Epithelial-mesenchymal transition (EMT) is a pathway implicated in cancer metastasis. [1]. This process involves the degradation of cell-cell and cell-extracellular matrix adhesions and the subsequent down-regulation of junctional proteins such as E-cadherin [1], [2]. Cells undergo a re-organization of the cytoskeleton and production of the type III intermediate filament vimentin [3]. These alterations are associated with a change in cell shape, from an epithelial to a mesenchymal or fibroblast-like morphology [4], [5].

Cancer cells are dependent upon extracellular cues from the tumor microenvironment [6], such as epidermal growth factor (EGF), which can promote breast cancer cell migration [7]. Goswami *et al* [8] have described an *in vivo* paracrine loop whereby colony-stimulating-factor-1 (CSF-1) expressing cancer cells recruit tumor-associated macrophages, which then secrete EGF, promoting cancer cell elongation and migration. *In vitro* some cell lines undergo EMT in response to EGF stimulation [4], such as the human breast cancer cell line MDA-MB-468.

Once converted to a migratory phenotype, cancer cells face a new set of environmental challenges. For example, the circulatory system and secondary tumor microenvironment may not be conducive to cell growth and survival. Cellular remodeling occurring as a consequence of EMT, whereby cells have altered responses to agents in the circulatory system or secondary tumor site, could be advantageous for the process of metastasis [9], [10].

A remodeling of cells, the consequence of which is an altered response to external stimuli, occurs in vascular smooth muscle cells, which convert from a contractile to a proliferative phenotype [11], [12]. Conversion of vascular smooth muscle cells to a proliferative phenotype is an important mechanism in vasculature repair but can also contribute to vascular disease [11]. The proliferative phenotype of vascular smooth muscle cells has alterations in the nature of responses to G-protein coupled receptor activators, such as angiotensin II, thrombin and vasopressin [13]. However, few studies have evaluated if analogous alterations in cell surface receptor-mediated signaling also occurs during the phenotypic switch associated with EMT in cancer cells.

Many cell surface receptors, including some receptor tyrosine kinases, G-protein coupled receptors, and ligand-gated ion channels signal via changes in cytosolic Ca^2+^ concentrations. Calcium is an important intracellular signaling molecule and regulates a diverse range of physiological and pathological processes [14], [15]. For example, the Ca^2+^-related proteins Orai1 and STIM1, important for store operated calcium entry pathways, are important in breast cancer cell migration and metastasis [16].

Two external stimuli that are important in breast cancer cells and elicit an intracellular Ca^2+^ response are serine proteases and adenosine 5′-triphosphate (ATP). Serine proteases activate the protease activated receptor (PAR) family of plasma membrane receptors [17]. PAR2 is a G-protein-coupled receptor that undergoes proteolytic cleavage and activation following exposure to the serine protease trypsin [18]. Activation of PAR2 triggers an intracellular signaling cascade downstream of phospholipase C activation, which results in the production of IP_3_ and the mobilization of Ca^2+^ from intracellular stores [19]. PAR2 silencing in the mesenchymal-like cell line MDA-MB-231 [20] inhibits cell migration [19]. The coagulant proteases VIIa and Xa are endogenous ligands for the PAR2 receptor; these coagulation proteins stimulate migration in human breast cancer cells via PAR2 activation [19]. ATP can also act as an external paracrine factor and tumor promoter, via its effects on P2X non-selective cation channels and P2Y metabotropic purinergic receptors [21]. Activation of these receptors results in elevation of cytosolic Ca^2+^ via influx (P2X) [22] and store-release (P2Y) mechanisms [23]. ATP is released in the micromolar concentration range in the tumor environment [24] and ATP increases proliferation of MCF-7 human breast cancer cells via Ca^2+^-dependent PI3K/Akt pathways downstream of P2Y_2_ and/or P2Y_4_ purinergic receptors [25].

In these studies we investigated whether EGF-induced EMT is associated with a remodeling of receptor isoforms to external stimuli. Consequent changes in intracellular Ca^2+^ signaling may help cells better meet the demands associated with metastasis.

## Results

### Changes in sensitivity to ATP

**Figure 1:**
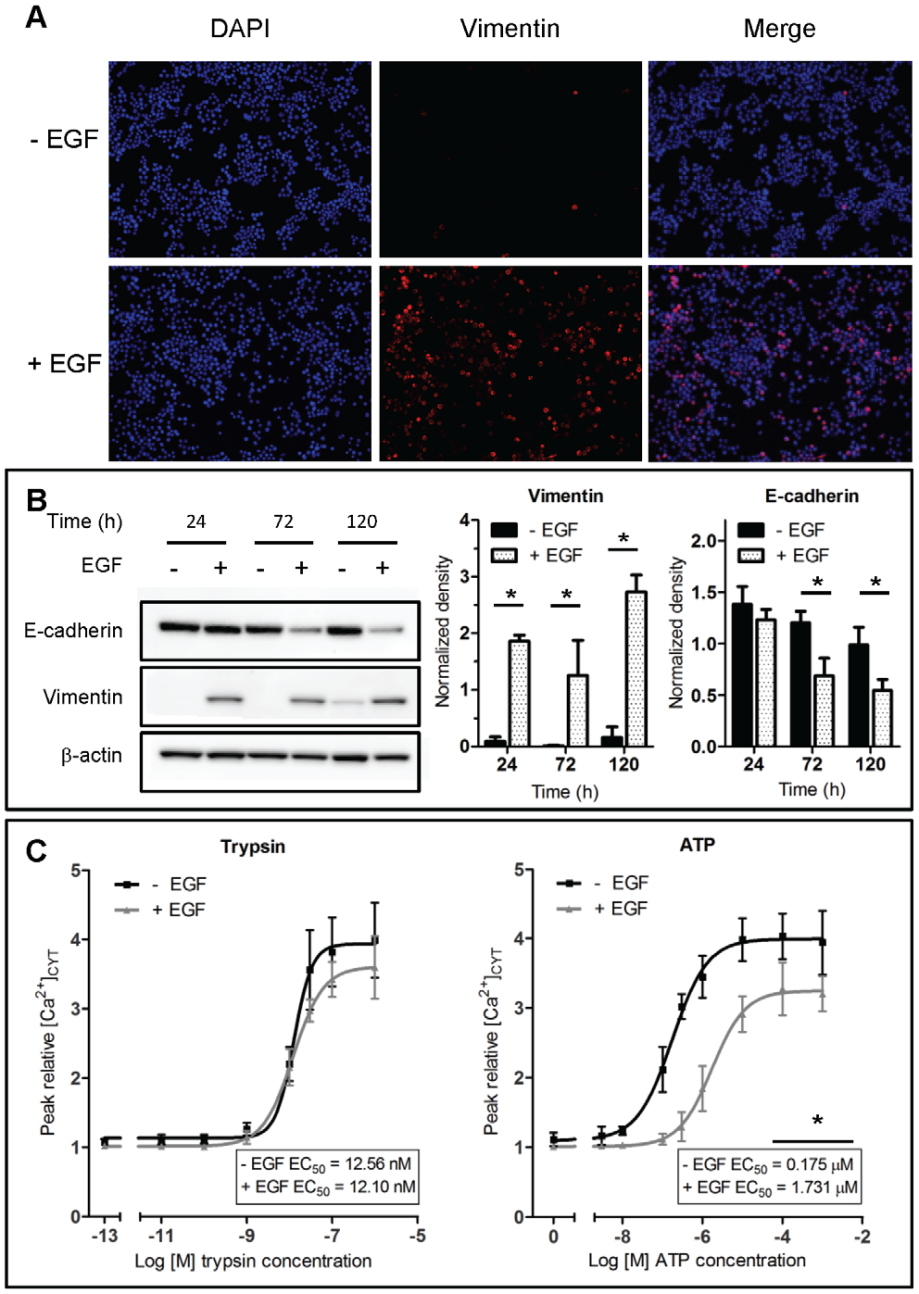
EGF-induced EMT and receptor-mediated Ca^2+^ signaling. MDA-MB-468 breast cancer cells were serum starved prior to treatment with EGF (50 ng/mL) or control for 24, 72 or 120 h as depicted. **A**, representative panel of immunofluorescence (IF) images showing vimentin expression (red) and DAPI nuclear staining (blue) following EGF stimulation (24 h). **B**, representative immunoblots for E-cadherin and vimentin protein after treatment with EGF (left) and pooled data (right) quantified relative to β-actin loading control. Pooled values represent mean ± S.D. for 6 pooled wells performed in triplicate in independent experiments. Statistical analysis was performed using two-way ANOVA with Bonferroni post-tests and * signifies *P*<0.05. **C**, assessment of [Ca^2+^]_CYT_ in MDA-MB-468 cells treated with EGF (24 h) following stimulation with various concentrations of either trypsin (PAR2 activation) or ATP (P2 receptor activation). Graphs represent the average dose response curves for measurement of peak relative [Ca^2+^]_CYT_ for 9 wells from 3 independent experiments and are shown ± S.D. Average EC_50_ values are shown inset and * represents the significance for EC_50_ values, *P*<0.05, unpaired t-test.

As previously described [4], MDA-MB-468 cells treated with EGF (50 ng/mL) had elevated levels of the mesenchymal marker vimentin after 24 h (Fig. 1 A & B) and a gradual decrease in the epithelial protein E-cadherin after 72 h (Fig. 1B). We also assessed the effect of EGF (50 ng/mL, 24 h) on Ca^2+^ signaling in MDA-MB-468 cells. While we saw no significant difference in the potency for PAR2 activation with trypsin we did observe a 10-fold statistically significant (*P*<0.05) shift in the potency for ATP compared to control cells (EC_50_ 1.731 µM and 0.175 µM, respectively) (Fig. 1C). This suggests that EGF can induce specific changes in the response to some extracellular stimuli including ATP. To investigate this effect further we examined the differential response and mechanism of ATP signaling associated with EGF-mediated EMT in MDA-MB-468 breast cancer cells.

### Changes in the nature of ATP-induced Ca^2+^ transients

**Figure 2:**
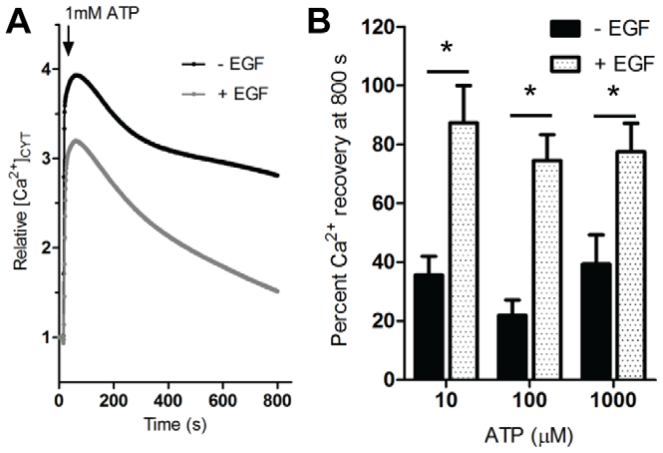
Effect of EGF treatment on the Ca^2+^ response to ATP stimulation. MDA-MB-468 cells were serum deprived and treated with or without EGF for 24 h as depicted. **A,** average [Ca^2+^]_CYT_ transient in cells stimulated with 1 mM ATP. **B,** [Ca^2+^]_CYT_ was assessed using 10 µM, 100 µM and 1 mM of ATP. Percent [Ca^2+^]_CYT_ recovery at the end of the assay (800 s) is shown for each concentration of ATP and represent the averages ± S.D. of 9 wells from 3 independent experiments. Statistical analysis was performed using two-way ANOVA with Bonferroni post-tests; * signifies *P<*0.05.

In addition to a change in agonist potency we observed a significant difference in the nature of the Ca^2+^ profile associated with ATP stimulation. Treatment with EGF for 24 h altered the post-peak decay kinetics in MDA-MB-468 cells stimulated with a range of ATP concentrations (Fig. 2). Cells exposed to EGF exhibited a faster return to baseline cytosolic Ca^2+^ levels than those in the absence of EGF.

**Figure 3:**
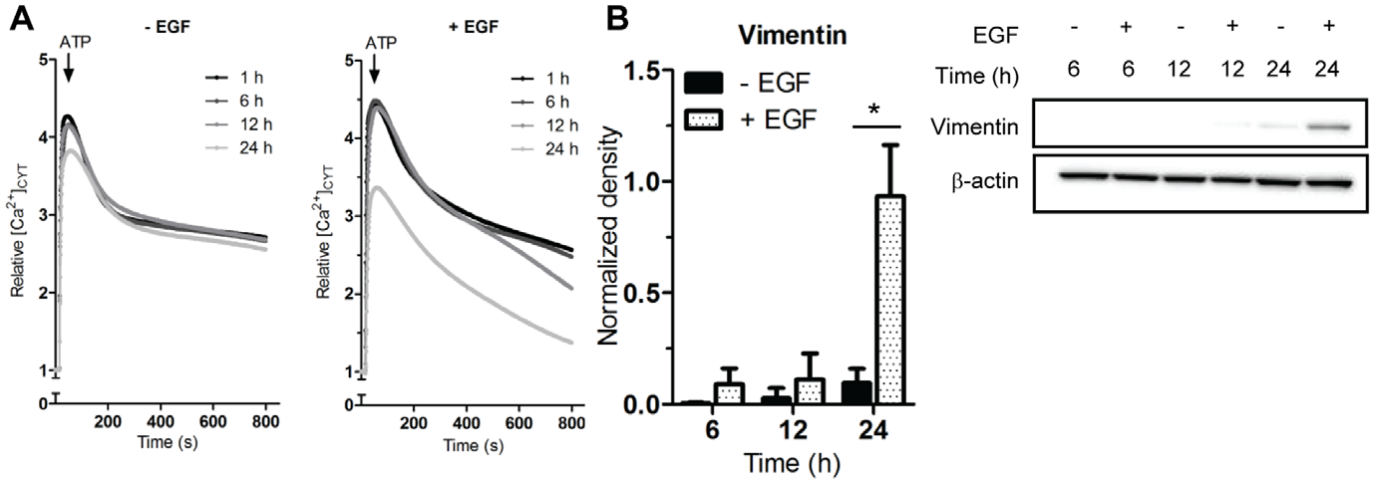
Expression of vimentin protein and functional alterations in the response to ATP. A, MDA-MB-468 cells were serum starved and treated with EGF for 1, 6, 12 and 24 h prior to measuring [Ca^2+^]_CYT_ with 1 mM ATP. Data are shown as the average relative [Ca^2+^]_CYT_ from 9 wells from 3 independent experiments. B, Representative immunoblot for vimentin protein after EGF treatment (left) and pooled data (right) normalized to the β-actin loading control. Values represent the mean ± S.D for 6 pooled wells from 3 independent isolations. Statistical analysis was performed using two-way ANOVA and Bonferroni post-tests; * *P*<0.05.

To assess the time dependence of this effect MDA-MB-468 cells were treated with EGF for 1, 6, 12 and 24 h prior to analysis of ATP-mediated increases in [Ca^2+^]_CYT_ (Fig. 3A). At 1 and 6 h post EGF treatment, the ATP-induced Ca^2+^ transients were unchanged and similar to those of control cells. However, modest alterations in the decay kinetics were apparent as early as 12 h following EGF exposure. At 24 h pronounced differences in the peak relative [Ca^2+^]_CYT_ and decay kinetics of the [Ca^2+^]_CYT_ transient mediated by ATP were evident; this effect corresponded to a significant increase in vimentin protein expression (Fig. 3B).

### EGF-induced alterations in the ATP response are not a consequence of the loss of cell-cell adhesion associated with EMT

**Figure 4:**
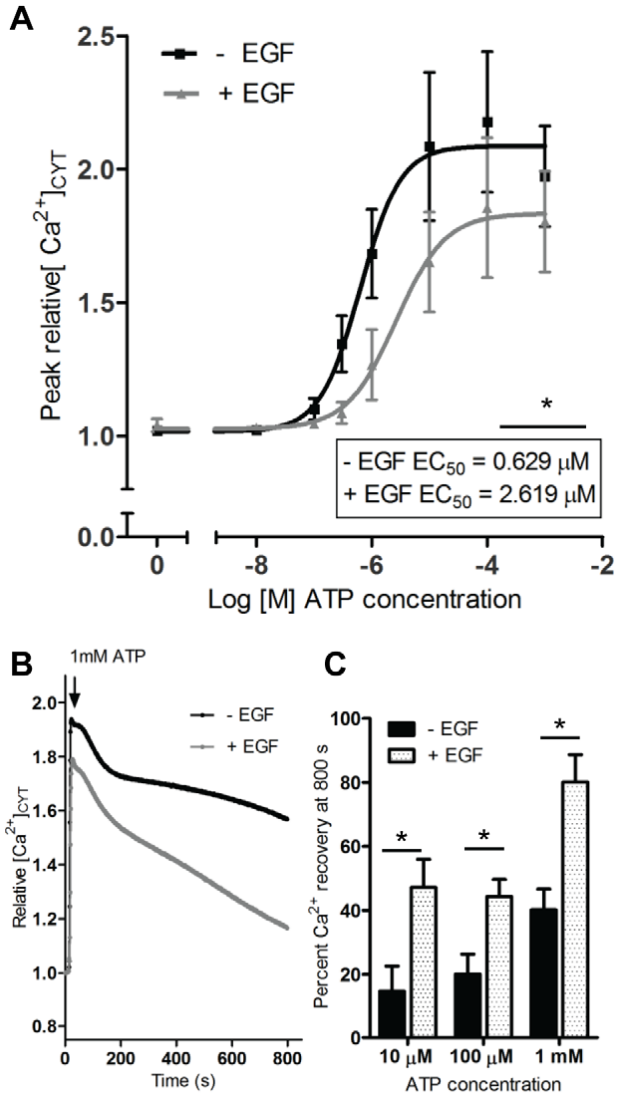
ATP signaling in non-adherent MDA-MB-468 cells following EGF treatment. A, assessment of [Ca^2+^]_CYT_ in non-adherent MDA-MB-468 cells treated with EGF (24 h) following stimulation with various concentrations of ATP. Graphs represent the average dose response curves for measurement of peak relative [Ca^2+^]_CYT_ and are shown ± S.D. The average EC_50_ values are shown inset and * represents the significance for EC_50_ values; *P*<0.05, unpaired t-test. B, the average [Ca^2+^]_CYT_ transient for suspended cells stimulated with 1 mM ATP. C, Percent [Ca^2+^]_CYT_ recovery ± S.D. at the end of the assay (800 s) was assessed using 10 µM, 100 µM and 1 mM of ATP. Statistical analysis was performed using two-way ANOVA with Bonferroni post-tests; * signifies *P<*0.05. Values are representative of 12 wells from 3 independent experiments.

A defining feature of EMT is a change in cell morphology and loss of cell-cell contacts [26]. Given that gap junctions facilitate inter-cellular communication by permitting the passage of Ca^2+^ ions and IP_3_ between neighboring cells [27], alterations in the nature of the [Ca^2+^]_CYT_ signal elicited by ATP could be due to EGF-induced loss of inter-cellular communication. To assess this we measured [Ca^2+^]_CYT_ in non-adherent MDA-MB-468 breast cancer cells. In suspended cells EGF treatment produced the same change in the ATP dose response curve (Fig. 4A) and the nature of the [Ca^2+^]_CYT_ transient (Fig. 4B & C) as seen in the adherent cells (Fig. 2). This suggests that alterations in the response to ATP in cells treated with EGF are not a consequence of the loss of cell-cell contacts associated with EMT.

### EGF induces a switch in the purinergic receptor profile of MDA-MB-468 breast cancer cells

**Figure 5:**
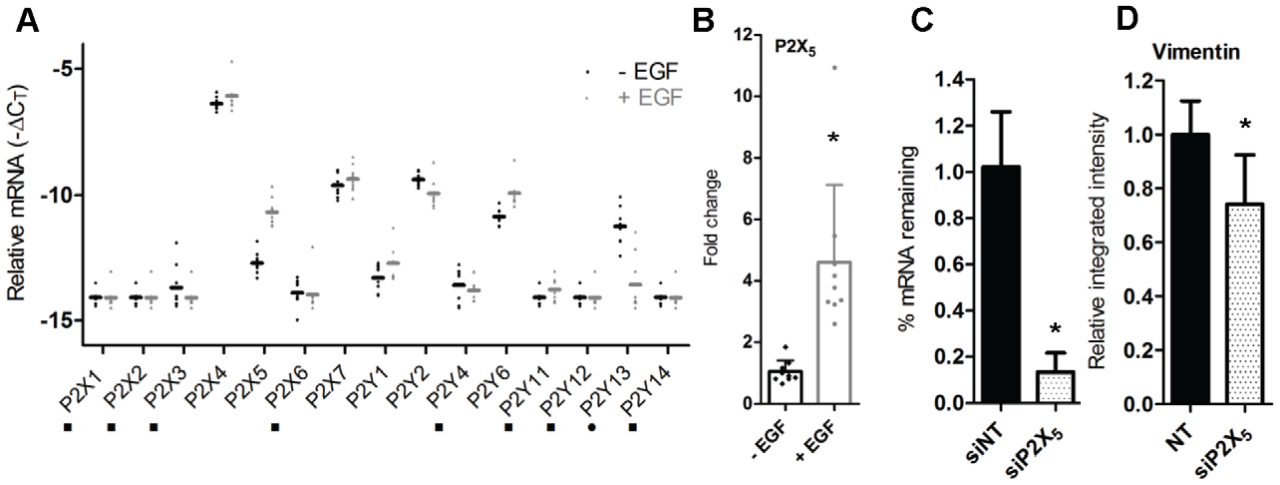
Effect of EGF on the transcriptional profile of purinergic receptors in MDA-MB-468 cells. Serum deprived MDA-MB-468 cells were treated with EGF or control for 12 h prior to RNA isolation and real-time RT-PCR analysis. A, −ΔC_T_ values of all P2 receptors studied. Samples with a C_T_ outside the limit of detection were assigned a value of 35 (▪ signifies target registered above the limit of detection (C_T_>35) in one or more samples for both EGF and control treatments; • denotes target was above the limit of detection in EGF samples only). B, Quantitation of alterations in P2X_5_ mRNA following EGF treatment (12 h). C, Knockdown efficiency of P2X_5_ siRNA (siP2X_5_) relative to the non-targeting control (siNT) was assessed using real time RT-PCR; representative of 5 wells from 3 independent experiments. D, EGF-induced vimentin protein expression (IF) in MDA-MB-468 cells treated with siNT or siP2X_5_. Results are representative of 9 wells from 3 independent experiments (unless otherwise specified) and are shown with S.D. (* *P*<0.05, unpaired t-test).

Another possible explanation for changes in ATP-mediated Ca^2+^ signaling may be due to alterations in the purinergic receptor profile in MDA-MB-468 cells as a consequence of EGF-stimulation. To investigate if changes in the transcription of purinergic receptors precede EMT, we analyzed the expression of a bank of purinergic receptors using real-time RT-PCR. Seven P2X (P2X_1–7_) and eight P2Y (P2Y_1, 2, 4, 6, 11–14_) receptor isoforms were studied in MDA-MB-468 cells stimulated with EGF. Changes in transcription were assessed 12 h post EGF-treatment, as alterations in gene transcription are expected to precede functional responses such as changes in ATP-induced Ca^2+^ signaling and vimentin protein induction. EGF-mediated EMT was confirmed by assessment of vimentin protein expression for all samples at 24 h (data not shown). Fig. 5A shows the relative levels of purinergic receptors in MDA-MB-468 cells in the absence and presence of EGF treatment. In both groups high levels of P2X_4_ were detected, which was not altered by EGF. In both treatment groups low to undetectable levels of mRNA were seen for P2X_1_, P2X_2_, P2X_3_, P2X_6_, P2Y_4_, P2Y_11_, P2Y_12_, and P2Y_14_.

However, treatment with EGF did induce a switch in the suite of purinergic ATP receptors including a 2.1-fold increase in P2Y_6_ mRNA and a 2.6-fold decrease in P2Y_13_ mRNA expression (Fig. 5A). The greatest induction of a purinergic receptor upon EMT induction was seen for P2X_5_, where EGF induced a 4.6-fold increase (Fig. 5B); suggesting that elevated P2X_5_ may be a characterizing feature of the metastatic phenotype of some breast cancer cells.

Given the magnitude of the increase in P2X_5_ in our model of EMT, we assessed the consequence of P2X_5_ knockdown on EGF-induced vimentin expression. We obtained a greater than 80% knockdown of P2X_5_ mRNA in cells transfected with P2X_5_ siRNA (siP2X_5_) relative to the non-targeting siRNA control (siNT) (Fig. 5C). Inhibition of P2X_5_ was associated with a modest but significant (*P*<0.05) decrease in EGF-induced vimentin protein expression (Fig. 5D).

P2X_5_ mRNA is significantly up-regulated in breast cancer cell lines with mesenchymal characteristics and aggressive basal-like clinical breast cancer samples.

**Figure 6:**
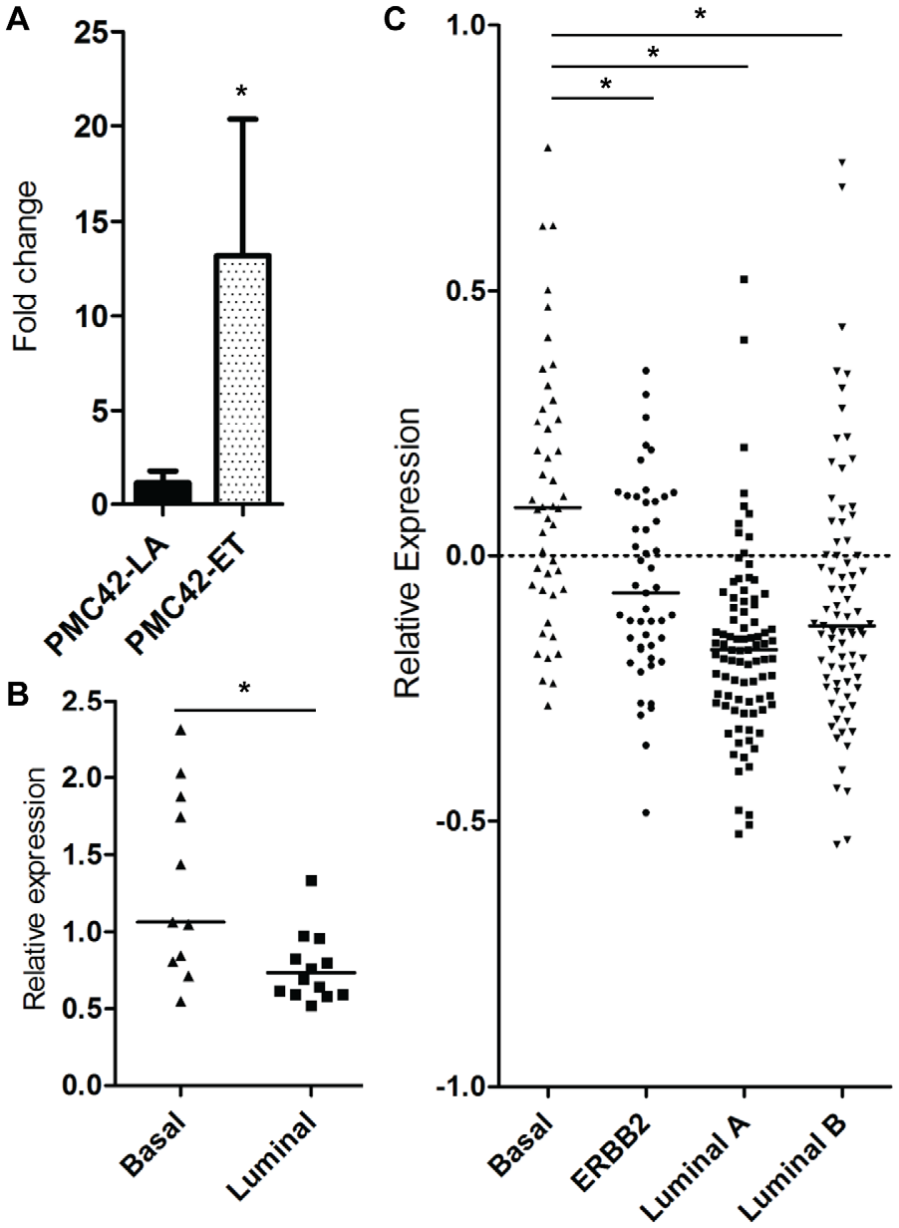
Expression of P2X_5_ in breast cancer cell lines and clinical samples. A, quantitation of P2X_5_ mRNA in PMC42-ET (mesenchymal) breast cancer cells relative to PMC42-LA (epithelial-like) breast cancer cells; * *P*<0.05, unpaired t-test. B, Relative P2X_5_ expression was examined using microarray data from 24 human breast cancer cell lines classified as luminal or basal via transcriptional profiling. A Mann-Whitney test was used for assessing statistical significance (*P<0.05). C, Evaluation of P2X_5_ levels in 264 human breast cancer samples representing four transcriptional subtypes. Statistical analysis was performed using Kruskall-Wallis test with Dunn's post-test and * signifies *P*<0.05. Horizontal lines represent the median value for each cluster.

To investigate the significance of P2X_5_ in breast cancer we examined its expression in other models of EMT and in clinical samples. We first compared P2X_5_ expression between the mesenchymal-like PMC42-ET breast cancer cell line and a derivative sub-line PMC42-LA, which expresses epithelial-like markers [28]. P2X_5_ had a 13-fold higher expression in PMC42-ET cells relative to PMC42-LA (Fig. 6A), further indicating an association with P2X_5_ and the metastatic phenotype. To investigate the distribution of P2X_5_ in a panel of breast cancer cell lines of known transcriptional subtype, we queried a microarray database of 24 human breast cancer cell lines for P2X_5_ [29]. P2X_5_ was significantly enriched in basal-like breast cancer cell lines compared to those of luminal origin (Fig. 6B). To determine the potential clinical relevance of this cell line data, we then examined a microarray gene expression dataset of 264 human breast cancer cases [30]. As we found with the cell lines, P2X_5_ was significantly overexpressed in tumors of the basal subtype compared to the luminal A and B subtypes and ERBB2+ tumors (Fig. 6C). Of the transcriptionally distinct breast cancer subtypes defined by Perou et al [31], basal-like breast cancers are highly aggressive, difficult to treat, and prone to metastasis. Furthermore these cancers have been linked to EMT and are transcriptionally akin to mesenchymal cells [32].

## Discussion

Metastasis represents a major cause of mortality in women with breast cancer [33], with EMT being increasingly investigated in this context. Relatively few studies have investigated changes in cell surface receptors that occur as a consequence of EMT, despite the altered extracellular signals that would be encountered by a cell as it metastasizes [34]. Using a model of EGF-induced EMT we investigated two cell surface receptors that signal via alterations in cytosolic calcium. Here we report a significant change in the potency of ATP-mediated Ca^2+^ signaling. This was not due to an overarching change in cell signaling cascades as there was an absence of a similar change observed in cells associated with PAR2 activation. In addition to an altered potency of ATP, a change in the suite of purinergic receptors was associated with EGF-induced EMT.

Alterations in responses to ATP as a consequence of EMT may be reflective of purinergic receptor-regulated processes important in tumor progression. ATP released via necrosis at the hypoxic core of solid tumors, may serve as an important paracrine signal in the tumor microenvironment [24]. Indeed, ATP signaling via ionotropic P2X and metabotropic P2Y receptors regulates a range of cellular events including proliferation, differentiation, apoptosis and invasion [21], [35]. The balance of these processes may therefore depend on the specific profile of cellular purinergic receptors expressed. PC3 and DU-145 hormone refractory prostate cancer cells, which have a similar expression profile (P2X_4,5,7,_ P2Y_1,2,4,6_) to estrogen and progesterone insensitive MDA-MB-468 breast cancer cells, undergo growth inhibition in response to ATP stimulation [36].

The kinetics and spatial characteristics of the Ca^2+^ transient dictate the activation of downstream signaling cascades and thus cellular responses to agonist activation [15]. Our observation of a change in the nature of the ATP-mediated cytosolic calcium transient in addition to the rightward shift in the dose response curve in cells treated with EGF is further evidence that altered responses to ATP are a likely consequence of EGF-mediated EMT.

In addition to altered Ca^2+^ signaling to ATP with EMT, we also report a change in the profile of purinergic receptors during the transformation from an epithelial to a mesenchymal-like phenotype. This change in the suite of purinergic receptor transcription was marked by a significant increase in P2X_5_, P2Y_6_ and a decrease in P2Y_13_ expression. A change in the purinergic receptor isoform profile is also seen during vascular remodeling [37]. Vascular smooth muscle cells transitioning from the contractile to proliferative (or synthetic) phenotype have reduced P2X_1_ levels and an increase in P2Y_1_ and P2Y_2_ mRNA expression [38]. Our results support that a remodeling of purinergic receptor transcription occurs as a consequence of EMT although different purinergic receptors are involved.

We investigated the potential significance of an increase in P2X_5_ purinoceptors during EGF-induced EMT, as this isoform was associated with the greatest alteration in purinergic expression observed in this model. To study the consequence of changes in P2X_5_ expression, we adopted a siRNA gene silencing approach to knockdown P2X_5_ in this model of EGF-induced EMT. P2X_5_ silencing significantly reduced EGF-mediated induction of the EMT marker vimentin. P2X_5_ receptors form functional homomeric trimers or may assemble into heteromultimers with P2X_1_ subunits [39], [40]. As P2X_1_ mRNA was undetectable in this cell line and underwent no apparent change in transcription with EGF-stimulation, P2X_5_ subunits would most likely assemble into homomeric ion channels in this cell-based model. Functional P2X_5_ homomeric channels are permeable to calcium and additionally display significant permeability to chloride ions and the large organic ion NMDG [39]. Alterations in chloride ion homeostasis occur in glioma cells and correlates with the invasive phenotype [41].

P2X_5_ activation with ATP inhibits the proliferation of skeletal muscle satellite cells and a role for P2X_5_ in the inhibition of cancer cell proliferation is proposed [42]. Cancer cells at the invasive front of solid tumors show a reduction in cell proliferation, coinciding with an increase in cell migration and invasion [43], [44]. A reduction in proliferation following ATP-mediated P2X_5_ activation may be an important mechanism in the switch from an epithelial (proliferative) to a mesenchymal (migratory) phenotype during the process of EMT [45].

To determine if alterations in P2X_5_ transcription may be a characterizing feature of some breast cancer cells associated with a more mesenchymal phenotype we examined the level of P2X_5_ expression in a breast cancer cell line with epithelial characteristics (PMC42-LA) compared to the parental mesenchymal cell line PMC42-ET [28]. P2X_5_ was enriched in the mesenchymal phenotype. This prompted us to investigate P2X_5_ expression in a bank of human breast cancer cell lines classified as luminal or basal by transcriptional profiling. P2X_5_ was upregulated in the more invasive basal-like cell lines compared to luminal-like cell lines. Assessment of P2X_5_ expression using microarray data from 264 human breast cancer samples classified as luminal (A/B), ERBB2+ or basal, indicated that P2X_5_ is significantly upregulated in the basal subset of clinical breast cancer samples compared to all other subtypes. The basal molecular subtype represents a subset of cancers that often express EMT-associated markers [32], have a poor clinical prognosis and are often associated with preferential metastasis to the lung and brain [31], [46]. The recently identified claudin-low intrinsic subtype of breast cancers has similarities to basal-like breast cancers, are triple negative and are enriched with EMT markers [47]. Moreover, gene expression profiles of the mesenchymal phenotype in breast cancer cell lines shows significant overlap with highly malignant breast cancer stem cells isolated from clinical subjects [48]. Future studies could further explore the role of P2X_5_ in the mesenchymal phenotype by characterizing expression in the claudin-low subset of breast cancers and malignant breast cancer stem cells. Future studies assessing the role of all the purinergic receptors altered in MDA-MB-468 as a consequence of EMT on intracellular calcium signaling and vimentin protein induction would also be valuable.

In conclusion, the induction of EMT by EGF in MDA-MB-468 breast cancer cells is associated with alterations in the calcium signaling response to ATP and results in a cellular phenotype with an altered transcriptional profile of purinergic receptors, in particular an upregulation of P2X_5_. Inhibition of P2X_5_ reduces expression of the EMT marker vimentin and its increased expression correlates with breast cancer cells that are associated with a more mesenchymal phenotype.

## Materials and Methods

### Cell culture and EGF treatment

MDA-MB-468 human breast cancer cells [4] were maintained in Dulbecco's Modified Eagle's Medium (D6546) supplemented with 10% fetal calf serum (FCS), L-glutamine (4 mM), penicillin 100 U/mL and streptomycin 100 µg/mL (Sigma Aldrich). To induce EMT, MDA-MB-468 cells were deprived of serum (0.5% FCS) for 24 h and stimulated with EGF (50 ng/mL; Sigma Aldrich) as previously described [4]. PMC42-ET and –LA human breast cancer cells [28], [49] were maintained in Roswell Park Memorial Institute (RPMI)-1640 Medium (R8757, Sigma Aldrich) supplemented with 10% FCS. Cultures were maintained in a humidified incubator (37°C, 5% CO_2_) and were routinely screened for mycoplasma contamination.

### Immunofluorescence

MDA-MB-468 cells were seeded at 3×10^4^ cells per well in 96-well black-walled imaging plates (BD Biosciences). After EGF treatment cells were fixed with methanol-acetone (1∶1). Mouse anti-vimentin V9 Cy3-conjugated antibody (C9080, Sigma Aldrich) was diluted 1∶400 in phosphate buffered saline supplemented with BSA (1% (w/v)) and incubated at 4°C overnight [49]. Nuclear staining was performed using DAPI (400 nM; Invitrogen) and incubated at room temperature for 1.5 h. Images were acquired using an ImageXpress Micro automated epifluorescence microscope (Molecular Devices Corporation).

### Immunoblotting

Cell extracts were harvested as previously described [50] using lysis buffer supplemented with protease inhibitors and phosphatase inhibitors (Roche Applied Science). For gel electrophoresis, samples were prepared using a reduced denatured protocol in lithium dodecyl sulphate (LDS) 4× sample buffer (Invitrogen). Approximately 20 µg of protein was loaded per well into a 4–12% bis-tris gel (Invitrogen). The separated proteins were transferred onto a polyvinylidene fluoride (PVDF) membrane (Invitrogen). Mouse anti-vimentin V9 antibody (V6389, Sigma Aldrich) was diluted to 1∶750 [49] and mouse anti-E-cadherin (a kind gift from Professor Alpha Yap, The University of Queensland, Australia) was used 1∶100. Anti-mouse horseradish peroxidase-conjugated secondary antibody (170–6516, BioRad) was used at 1∶10000. All antibodies were prepared in 5% skim milk powder in PBST (0.1% Tween-20). Images were acquired on a VersaDoc Imaging System (BioRad) and quantified using ImageJ (v1.43u for Windows, National Institutes of Health, USA). Protein density was normalized to the β-actin (A5441, 1∶10000, Sigma Aldrich) loading control.

### Measurement of intracellular Ca^2+^

Calcium assays were performed with a fluorometric imaging plate reader (FLIPR^TETRA^, Molecular Devices Corporation) using the no-wash PBX Ca^2+^ Assay Kit (BD Biosciences) as previously described [51]. For the measurement of intracellular Ca^2+^ in adherent cells, MDA-MB-468 cells were seeded at 3×10^4^ cells per well in 96-well black-walled imaging plates (Corning) and treated with EGF. For the measurement of intracellular Ca^2+^ in suspended cells, cells were treated with EGF, trypsinized and resuspended in DMEM containing FCS (0.5%), BSA (0.3%) and Fluo-4 AM Ca^2+^ indicator (2 µM). Cells were incubated in centrifuge tubes for 60 min at 37°C. Following dye uptake, cells were centrifuged and resuspended in physiological salt solution (PSS) buffer. Suspended cells were seeded in 96-well black-walled plates at a density of 6.15×10^4^ cells per well. Intracellular Ca^2+^ measurements were performed with an excitation intensity of 470–495 nm and a 515–575 nm emission filter. Fluorescent values were normalized to the starting fluorescence and are expressed as relative Ca^2+^_[CYT]_.

### Real time RT-PCR

Cells were plated at 8.5×10^5^ cells per well in a 6-well plate (P2 purinergic screen) or from a 96 well plate (P2X_5_ studies) and cells were treated with EGF. Total RNA was isolated using the RNeasy kit (Qiagen) as per the manufacturer's instructions. RNA was reverse transcribed (Qiagen Omniscript RT Kit) and amplified using TaqMan Custom Array plates spotted with a selection of purinergic receptor assays (Table S1) and TaqMan Universal Master Mix (Applied Biosystems). Reactions were cycled with universal cycling conditions and a StepOnePlus Real Time PCR System (Applied Biosystems). Relative quantification was determined with reference to 18s ribosomal RNA and analyzed using the comparative C_t_ method as previously described [52].

### Gene expression microarray

Breast cancer cell lines were cultured in 2D and analyzed using Affymetrix microarrays as previously described [29]. This panel of cell lines included 13 luminal-type (600MPE, BT474, BT483, CAMA1, MCF7, MDA-MB-361, MDA-MB-415, MDA-MB-453, SKBR3, T47D, UACC812, ZR751 and ZR75B) and 11 basal-type cell lines (BT549, HMT3522-S1, HMT3522-T4-2, HCC1500, HCC1569, HCC70, HS578T, MCF12A, MDA-MB-231, MDA-MB-436 and MDA-MB-468). All cell lines were acquired from ATCC, except HMT3522-S1 [53] and HMT3522-T4-2 [54] which are available from Sigma Aldrich. P2X_5_ expression was assessed using the probe 210448_s_at. We also mined microarray data from 295 breast cancer cases [30]. We excluded the 31 samples in this dataset annotated as “normal-like” as these contain a substantial proportion of contaminating non-neoplastic cells, and focused on the 264 tumors annotated as either basal, ERBB2+ and luminal A or B. MIAME-compliant raw microarray data are available from the following sources: ArrayExpress E-TABM-244 (cell lines) and NCBI GEO GSE2845 (tumors).

### siRNA transfection

For all siRNA experiments, cells were cultured in antibiotic free media. MDA-MB-468 cells were plated at a seeding density of 5×10^3^ cells per well in a 96-well plate. siRNA transfection was performed with Dharmacon ON-TARGET*plus* SMARTpool™ siRNA (100 nM), comprising a pool of 4 siRNA sequences rationally designed with dual strand modification and use of an algorithm to reduce seed region matches. DharmaFECT4 transfection reagent was used (0.1 µL/well) as per the manufacturer's instructions. Cells were serum starved 48 h post-transfection. At 72 h post-transfection cells were stimulated with EGF or control for 24 h prior to fixing and staining for vimentin expression. For all studies, successful knockdown (>80%) was confirmed at the transcriptional level 72 h post-transfection. The following Dharmacon siRNAs were used in this study: On-TARGETplus SMARTpool™ nontargeting siRNA (D-001810-10-05) and On-TARGETplus SMARTpool™ P2X_5_ siRNA (L-006286-00-0005).

### Statistical analysis

Statistical analysis was performed using GraphPad Prism version 5.04 for Windows unless otherwise stated. Specific statistical tests used are described in each figure legend.

## Supporting Information

Table S1 **TaqMan Assay IDs for the panel of P2 purinergic assays used for real-time RT-PCR.**
(PDF)
