# Supplementary material for: Remodeling of Purinergic Receptor-Mediated Ca2+ Signaling as a Consequence of EGF-Induced Epithelial-Mesenchymal Transition in Breast Cancer Cells
Source: PLoS One. 2011 Aug 5;6(8):e23464. doi: 10.1371/journal.pone.0023464 (PMC3151299; doi:10.1371/journal.pone.0023464)
Supplement: Table S1 — TaqMan Assay IDs for the panel of P2 purinergic assays used for real-time RT-PCR. (PDF) [file pone.0023464.s001.pdf]

Table S1: TaqMan Gene Expression Assays	
**Gene symbol**	**Assay ID**
**$P2X_1$**	Hs00175686_m1
**$P2X_2$**	Hs00247255_m1
**$P2X_3$**	Hs00175689_m1
**$P2X_4$**	Hs00175706_m1
**$P2X_5$**	Hs01112467_m1
**$P2X_6$**	Hs01003997_m1
**$P2X_7$**	Hs00175721_m1
**$P2Y_1$**	Hs00704965_s1
**$P2Y_2$**	Hs00175732_m1
**$P2Y_4$**	Hs00267404_s1
**$P2Y_6$**	Hs00602548_m1
**$P2Y_{11}$**	Hs01038858_m1
**$P2Y_{12}$**	Hs00224470_m1
**$P2Y_{13}$**	Hs03043902_s1
**$P2Y_{14}$**	Hs00208434_m1
